# The Trier Social Stress Test and the Trier Social Stress Test for groups: Qualitative investigations

**DOI:** 10.1371/journal.pone.0195722

**Published:** 2018-04-11

**Authors:** Olivier Vors, Tanguy Marqueste, Nicolas Mascret

**Affiliations:** Aix Marseille Univ, CNRS, ISM, Marseille, France; Universitat Wien, AUSTRIA

## Abstract

The Trier Social Stress Test (TSST) and its version for groups (TSST-G) are the gold standard for inducing acute psychosocial stress in human experimental settings and have been used in numerous studies since the early 1990s. The TSST and the TSST-G lead to effects on different physiological and psychological markers, such as salivary cortisol, anxiety, and emotional states. These effects were assessed with quantitative methods comparing pre-test and post-test measures with statistical analyses. But to date, no qualitative analyses have been conducted to examine the meaningful experience of participants during the TSST and the TSST-G. This study is the first to conduct qualitative investigations to further clarify the stressful experience of participants confronted with these procedures. Preliminary results showed as expected that the TSST and the TSST-G effectively induced psychosocial stress, with cortisol levels, cognitive anxiety, somatic anxiety, and arousal increases, and with pleasure and dominance decreases. At the qualitative level, the results evidenced that the two theoretically stressful components of the TSST and the TSST-G, namely social-evaluative threat and uncontrollability, were experienced as stress-inducing by the participants. Two case studies confirmed these findings. But the results also showed on one hand that psychosocial stress is a dynamic phenomenon, with important fluctuations throughout the tasks (mainly for the TSST-G); and on the other hand that despite the similar physiological and psychological responses between the TSST and the TSST-G evidenced by the literature, the experience of the participants was both similar and specific. Use of a qualitative method allowed us to provide a complementary point of view to understand the meaningful experience of participants during these stressful procedures, apprehending the dynamic of the subjective stress experience without disrupting it.

## Introduction

The development of validated methods to experimentally induce psychosocial stress has been a major research topic for many years. Several experimental psychosocial stress paradigms exist in the literature, such as the Socially Evaluative Cold Pressor Task [1], the computerized mental arithmetic task [2], or the Trier Social Stress Test (TSST [3]). But to date, no study using qualitative investigations has been conducted to further clarify the states experienced by the participants during these kinds of stressful tasks. Investigating how participants experience psychosocial stress induced by social-evaluative threat and uncontrollability is an interesting track to explore.

### Theoretical and empirical investigations on psychosocial stress

Psychosocial stress is caused by non-metabolically demanding tasks. Tasks with physical stressors (e.g., physical activity), physical-psychological stressor combinations (e.g., cold pressor) and biological challenge or placebo injection (e.g., caffeine) are not considered psychosocial stressors [4]. An extensive literature has shown that several psychological stressors may influence the hypothalamic-pituitary-adrenocortical (HPA) axis, which regulates the release of cortisol. In their meta-analysis on the relations between acute stressors and cortisol responses, Dickerson and Kemeny [4] showed that the effects of psychological stressors on HPA axis were discussed in the literature. Two psychosocial stressors were particularly studied: social-evaluative threat and uncontrollability. Social-evaluative threat occurs when an individual is or could be negatively judged by others [4]. According to the self-preservation theory [5], threats to an individual’s social self (i.e., social esteem, status, and acceptance) induce psychological and physiological responses to cope with these threats. The social self is highly related to others’ perceptions and humans seek to maintain their social self. In cognitive tasks, failure or poor performance would be considered as a lack of ability identified by others and therefore lead to a loss of social esteem and/or social status, because competence and intelligence are important aspects of self-identity. This social-evaluative threat often leads to an activation of the HPA axis and consequently a cortisol rise, and often also leads to psychological consequences such as increased anxiety [4]. Following the work of Lamarche et al. [6] on body image threats using self-preservation theory, providing insights through a qualitative approach to social-evaluative threat induced by validated stress protocols may be helpful to complete or clarify the evidence found with quantitative measures.

The second psychosocial stressor, uncontrollability, is the inability of a behavioral response to affect an outcome [7]. Consequently, an uncontrollable condition creates a context of forced failure because the individual’s efforts do not bring success in the task. The qualitative approach in the domain of work has shown that the study of uncontrollability is a promising route toward an evolution of traditional work satisfaction research [8]. To date, uncontrollability induced by some stress protocols has not been studied with a qualitative approach, whereas quantitative research is more focused on this form of psychosocial stress. For example, because nothing can specifically be done to avoid failure, uncontrollability may induce cortisol increases. While the animal literature provides strong support for this hypothesis, the human literature is inconclusive, with contrasted results [4]. Uncontrollability seems to induce cortisol responses only if an important goal is threatened, such as the preservation of the social self. Indeed, when task performance may be negatively judged by others and at the same time may not be controllable despite the individual’s best efforts, it is unlikely that his social self can be preserved, which activates the HPA axis, resulting in a significant cortisol rise. The meta-analysis conducted by Dickerson and Kemeny [4] clearly evidenced that both social-evaluative threat and uncontrollability were simultaneously necessary to induce the largest and the most reliable increases in cortisol in motivated performance tasks. While the effect sizes of the studies using one component alone are significant (*d* = .32 for uncontrollability, and *d* = .35 for social-evaluative threat), effect size was nearly three times as great (*d* = .92) when social-evaluation threat and uncontrollability were used simultaneously in the stressful task.

While several experimental paradigms induce psychosocial stress, Giles et al. [9] evidenced that the most robust effects on psychological and physiological measures in terms of intensity and duration are produced by the Trier Social Stress Test (TSST [3]). The TSST is the gold standard in human experimental stress and has been used in numerous studies [10]. The TSST, designed in the early 1990s, is an acute stressor that can be used as a valid and reliable experimental stress procedure [3]. In the standard TSST procedure, the participant is confronted with two consecutive tasks. The first one is a role-playing scenario in which s/he imagines s/he has applied for a job of his/her choice. After the 5-min speech, the second task is a mental arithmetic task (serial subtraction). The committee is composed of two or three absolutely neutral experimenters wearing white lab coats. The unknown experimenters are seated behind a table on which a wide-angle video camera, a microphone, and a timer have been placed. The participant is informed that the two experimenters are experts in nonverbal behavior analysis and his/her behavior will be videotaped and recorded for further analysis. The TSST is a single-subject method. The Trier Social Stress Test for Groups (TSST-G [11]) was recently designed for group testing, following the initial work of Childs, Vicini, and De Wit [12]. Six participants are separated by mobile dividing walls that restrict visual and social interaction. They face the same committee as in the TSST. After a 10-min preparation period, each participant performs the mock job interview for 2 min. Then the experimenters interrupt the first participant and request the next one to speak. The participants are not aware of the order in which they will be called. In the mental arithmetic task, each participant successively performs serial subastraction for 80 sec. In sum, the TSST-G is a validated simultaneous group version of the TSST which has been used in numerous studies (e.g. [13,14]). The TSST and the TSST-G lead to effects on different physiological and psychological markers (for a review, see [15]). For example, adrenaline increases following the TSST [16], whereas heart rate rises throughout the TSST and returns to pre-stress level a few minutes after the stressor cessation [12]. But salivary cortisol, a hormonal marker of anxiety, is certainly one of the most useful markers of the stress response with the TSST [10,15], since the literature has shown that it is responsive to the TSST, and salivary cortisol is easier to collect compared with blood sampling. Furthermore, the TSST increases anxiety [17], worsens negative mood [18], and modulates numerous forms of complex cognition [15]. The TSST-G induces a similar pattern of psychological, endodrine and cardiovascular stress responses to the TSST [11]. Without being exhaustive, these examples show that the TSST and the TSST-G lead to numerous physiological and psychological consequences.

The TSST includes, combines, and operationalizes social-evaluative threat and uncontrollability. It leads to significant physiological and psychological responses because (1) social-evaluative threat is characterized by the evaluative audience represented by the two experimenters, the video camera, and the microphone; (2) the committee members wearing white lab coats are presented as experts in behavior analysis, which emphasizes the importance of the task; (3) they are absolutely neutral, they do not smile or nod, and consequently they do not provide positive feedback or social support which might have preserved the social self; (4) the mock job interview and the mental arithmetic task create a context of forced failure; and (5) uncontrollability is included throughout the procedure: the room is unknown to the participants, they are not aware of the tasks that they will have to do, the duration of the preparation period is short, the mental arithmetic task is totally unanticipated, and the committee members are non-responsive to potential social interactions. The TSST-G shares all these components, but some of its specific characteristics lead social-evaluative threat and uncontrollability to be considered in a particular way. Social-evaluative threat is here represented by the committee members and the video camera, but also by the other participants. Although the six participants are separated by mobile dividing walls, each participant knows that the five others are present in the same room and that they will listen to him/her and judge his/her performance. Consequently, a negative social comparison is possible, inducing a high level of social-evaluative threat. Another form of uncontrollability is present in the TSST-G but not in the TSST: the participants do not know the order in which they will be called. Moreover, the order is different between the mock job interview and the mental arithmetic task. In sum, even if the two tasks have a shorter duration for each participant in the TSST-G (2 min for the interview and 80 sec for the mental arithmetic task) compared with the TSST (5 min for each task), the group format of the TSST-G leads to supplementary forms of social-evaluative threat and uncontrollability which provide similar physiological and psychychological responses compared with the TSST [11].

The theoretical framework, the meta-analysis of Dickerson and Kemeny [4], and many empirical results have confirmed that the social-evaluative threat and uncontrollability dimensions of the TSST and the TSST-G induce stimulation of the HPA axis and psychological effects, leading to cortisol increases, self-reported anxiety, and negative emotional states. Some examples of qualitative studies conducted on social-evaluative threat [6] or uncontrollability [8] showed that a qualitative approach would be likely to provide rich information missed through quantitative designs. But to the best of our knowledge, no qualitative investigations have been conducted during the TSST and the TSST-G to understand the dynamic of participants’ experience.

### Qualitative investigations of the dynamic of experience during the TSST and the TSST-G

Qualitative investigations are particularly helpful to describe and understand behaviors and experiences in a specific context (e.g. [19]). Studies using the TSST or the TSST-G were exclusively quantitative, measuring different physiological and psychological markers (e.g. [15]). These different markers validate the effects of those tests, but they cannot help us to understand what is experienced during the procedure. Qualitative data may provide knowledge about both experiment outcomes and participants’ experience during these experimental stressful tasks. The qualitative four-E approach [20–22] of an enactive phenomenological framework is useful for the present study mainly because it allows us to access actors’ significant experience. Significant experience is the meaning that the actor assigns to his actions in the situation, in accordance with the semiotic approach to cognition and action inspired by Peirce (1931–1935) [20–25]. This semiotic approach considers that human experience is composed of meaningful units like intention and perception, as used in this study. Thus, significant experience corresponds to the meaningful units that can be described, commented on, and shown by the participant. In this way, we may access participants’ experience through its meaningful dynamic [26], which affects physiological and psychological markers following the TSST and the TSST-G.

This enactive phenomenological framework presented by Rochat et al. [20] as a four-E approach examines, following Theureau’s tradition [21,22], human activity:

1. Embedded in the dynamic of the changing situation, i.e. considering the flow of action indivisible from its context [27]. This point of view allows us to access the dynamic of the participant’s action during the test situation.
2. Extended by artifacts, i.e. considering the action supported by objects or things of the environment [28]. This point of view allows us to understand what artifact of the TSST and the TSST-G supports the participant’s activity.
3. Embodied as recurrent sensorimotor patterns of perception and action, i.e. considering action and sensation as an inseparable whole [26,29]. This point of view allows us to access perception, sensation, sentiment, and emotion during the participant’s action.
4. Enacted by bringing forth a cognitive being’s world into an actor/environment coupling, i.e. considering that action and cognition emerge in the situation [26,30]. This point of view allows us to analyze what kind of action and cognition emerge during the TSST and the TSST-G situations.

From this perspective, the actor’s signification is constructed during action and can be revealed following a rigorous phenomenological method using a self-confrontation interview known as the enactive interview [21,22]. This qualitative approach produces a different point of view on the TSST and the TSST-G, because we can analyze the kind of action that emerged during the procedure, the signification of the action for the participants, the objects or tools supporting action, and the temporal dynamic of the experience.

A few recent studies have demonstrated the fruitfulness of this qualitative approach using this enactive phenomenological framework in the fields of sport (e.g. [24]), doping (e.g. [23]), work (e.g. [31]), or education (e.g. [25]). This framework is pertinent to study emotion and stress, as some studies have shown [32–34]. Without prejudging stress phenomena, those inductive studies analyze actors’ experience in different situations. For example, Ria et al. [33] studied teachers’ situated emotions. They analyzed the emergence circumstances of emotion, and they highlighted the emotional dynamic during teaching situations. Sève et al. [34] studied emotions experienced during high-stakes table tennis matches. They analyzed the dynamic of emotions during matches, and they showed relations between emotions and specific events. For example, “the pleasant or unpleasant tone of emotional content was linked to the set result and the interpretations of the unfolding situation. However, other elements of the competitive interaction (score configurations, judgments about the strokes performed) had a strong emotional coloration. Certain similar events (e.g., bad sensations during stroke performance) were frequently coupled with similar emotional content (e.g., displeasure).” ([34], p. 25). Some studies in this enactive phenomenological framework have managed to categorize the stressors and analyze how stressors are dynamically appraised [32]. By analyzing the experience of actors in situation, these studies access the temporal dynamic of emotional experience, and categorize recurring stressors shared by all the participants. With this enactive phenomenological framework, it may be heuristic to study the participants’ experience during the TSST and the TSST-G, which is difficult, if not impossible, with quantitative approaches. Studying the effects of time (pre-stress, post-stress) on physiological and psychological markers does indeed evidence that the TSST or the TSST-G are effectively stress-inducing when comparing all these variables from the beginning to the end of the stress procedures; but it does not provide information about what is happening during the stress procedures. Boesch et al. [13] used more precise measures than comparison between pre-stress and post-stress–at the end of the preparation period, at the end of the speech task, and at the end of the mental arithmetic task. But here too, the stressful experience during each task was not assessed. Schlotz et al. [35] proposed to repeat ratings of emotional states throughout the stress episodes to better represent the dynamics of subjective stress experience than pre-test/post-test assessments. But there is a risk that these repeated measures will disrupt the stress experience, thereby diminishing its effectiveness. Qualitative analyses as conducted in the present study are a “non-invasive” method to access the dynamic of the subjective stress experience. They provide a complementary perspective toward a better understanding of the significant experience of participants confronted with psychosocial stress, and of how social-evaluative threat and uncontrollability may be stressful and lead to physiological and psychological responses. For example, we intend to investigate social-evaluative threat throughout the TSST: is it stable due to the continued presence of the two experimenters, or is it dynamic, with temporary highs and lows? Furthermore, the theoretical framework of the TSST-G postulates that socio-evaluative threat is induced by both the committee members and the peers [11]. Do participants really experience these two forms of social-evaluative threat? Do they experience them in a consistent manner throughout the different steps of the TSST-G? We could also, for example, raise questions relating to the different levels of uncontrollability experienced by participants during the preparation period, the mock job interview, and the arithmetic task. In sum, this phenomenological analysis allows us to understand the emotional dynamic during the TSST and the TSST-G, which has never previously been done.

### The present study

The TSST is one of the most widely used tools and the gold standard to experimentally induce acute psychosocial stress [10]. The social-evaluative threat and uncontrollability dimensions included in the TSST lead to physiological and psychological consequences that are now widely known, even if further studies are required to pursue the investigations [15]. The present study uses a qualitative approach to examine in more depth the experience of participants confronted with the TSST or the TSST-G. Using this kind of approach to assess participants’ experience during these experimental stressful tasks is a promising way to better understand complex phenomena such as psychosocial stress and to further investigate what makes the TSST and the TSST-G stressful for participants.

## Materials and methods

### Participants

Eighteen men (20.22 ± 2.41 years with a range of 18–24 years; Body Mass Index of 23.71 ± 2.40 kg/m^2^) voluntarily participated in the study. Exclusion criteria were levels of chronic stress (French version of the Perceived Stress Scale [36]), psychological distress (French version of the General Health Questionnaire [37]), reported medical illness, history of endocrine disorder, medication intake, drug abuse, smoking more than five cigarettes a day, and drinking more than two glasses of alcohol a day. The study was approved by the institutional board of the faculty of sports sciences of Marseille and followed the ethics recommendations of Aix-Marseille University. Participants were informed that they could stop their participation at any time. They provided written informed consent following the requirements of the Declaration of Helsinki [38]. After completion of the experiment, all the participants were debriefed and thanked for their participation.

### Procedure

Nine men were confronted with the TSST condition and the nine others with the TSST-G condition. The assignment of condition was randomized. Experimental sessions for the TSST were conducted between 10:30 and 17:00 and took about 55 minutes (approximately 15 minutes for the TSST and 40 minutes for the enactive interview). Specific instructions were provided to the participants when they were recruited and were repeated by email and SMS with acknowledgment of receipt two days before the experiment. Participants in the morning sessions were instructed to wake up at least three hours before their appointed time in order to control the cortisol awakening response [39]. Practicing sport, consuming alcohol, and taking medication were not allowed in the twelve hours preceding the experiment. Eating, drinking (except water), brushing teeth, and smoking were not allowed from one hour before the beginning of the experiment. Participants were welcomed individually in the laboratory by experimenters who were not involved in the TSST and compliance with instructions was first checked. A questionnaire was completed and the first saliva sampling was done. Then participants entered the room dedicated to the TSST. After approximately 15 minutes, the participants left the room and a second saliva sampling and a second questionnaire were completed. Finally, each participant attended an enactive interview to access his experience.

The procedure for the TSST-G condition was almost the same as the one that was conducted with the TSST. Consequently, we only present the differences between the two procedures. Experimental sessions started in the afternoon between 14:00 and 17:00. The TSST-G lasted approximately 25 minutes. Due to the group format of the TSST-G, the 70-min interviews used in the qualitative part of the experiment could not be carried out immediately after the TSST-G for each participant, contrary to the TSST condition. They took place during the following week. Two TSST-G sessions were conducted, with five participants in the first session and four participants in the second one. To be in line with the initial TSST-G procedure [11], one confederate in the first session and two confederates in the second session played the participants’ role in order to have six participants in each session and to compare their experience during the TSST-G with qualitative investigations for a comparable duration. The TSST-G procedure is also slightly adaptable. For example, the preparation period may be 2 min long and the calculation task 2 min long for each participant [13], whereas in the initial procedure the preparation period was 10 min long. A recent review [40] showed that shortening speech preparation time did not influence cortisol responses in any way. Its authors suggested that reducing speech preparation time can be one TSST element useful for reducing the burden for participants as well as laboratory logistics. Consequently, the preparation time was 3 min in the present study.

### Physiological and psychological measures

The main purpose of the following physiological and psychological measures was to establish whether the TSST and the TSST-G had indeed induced psychosocial stress between the beginning and the end of the two protocols. In the present study, these measures were only considered manipulation checks in order to then conduct qualitative analyses. The TSST and the TSST-G were not compared with quantitative measures, because this was not the aim of our study–focused on qualitative investigations–and because the effects of the TSST and the TSST-G on different physiological and psychological markers were similar despite procedural differences (see also [11]). But we hypothesized that these procedural differences may lead to identification of specificities in qualitative analyses. Moreover, some of the single-TSST sessions took place in the morning; consequently no quantitative comparison between cortisol levels in the TSST and the TSST-G was carried out without controlling session time of day.

Our sample size could be considered rather small for a quantitative study and it was exclusively composed of men. The inherent constraints of qualitative approaches led us to focus on the 18 participants’ experience in order to study in depth the psychosocial stress they had felt. Moreover, our sample size was in line with recent publications using such a method (e.g. [20,24,31,32,41–44]). Only men were recruited in the study because oral contraceptives and the period of menstrual cycle may influence women’s cortisol levels [45]. Women could be included in further studies in order to establish whether they experienced the social-evaluative threat and the uncontrollability of the TSST and the TSST-G in the same way as men. Furthermore, cortisol and psychological variables were assessed twice: first, one minute before the TSST, then one minute after the TSST. The stress response is a dynamic phenomenon with anticipation, acute response, and recovery, and usually several repeated measures of cortisol levels (6 to 9) were conducted in experimental studies. The literature has shown that the peak in salivary cortisol levels occurs approximately 10 min after the cessation of the stressor [15], whereas anxiety and negative emotional states decrease more quickly than salivary cortisol levels [46]. A recent review of the effects of protocol variations on cortisol responses [40] showed that the number of post-TSST samples ranged from 1 to 11, and that the most commonly sampled times were immediately after the end of the TSST, 10–15 min, 20–25 min, and 30–35 min after. Our study focused on the qualitative aspects of the stress experienced by the participants during the TSST or the TSST-G and, bearing in mind the small sample size, physiological and psychological measures were only manipulation checks confirming that the stress protocols were indeed stress-inducing. Consequently, we retained two measurement points to evidence possible effects of time for cortisol levels, state anxiety, pleasure, arousal, and dominance between the beginning and the end of the TSST or the TSST-G. If these changes are significant, qualitative investigations can be conducted from a stronger basis than only making qualitative investigations. Furthermore, participants’ experience was not examined during the period preceding the TSST or the TSST-G and during the recovery period after the stressful tasks. Physiological and psychological measurement points during these two periods were not necessary, since the present study only focused on the subjective experience perceived during the stressful tasks. They could be considered in future studies exploring participants’ experience during the different stages of the stress response (anticipation, acute response, and recovery) induced by the TSST or the TSST-G, with a bigger sample size, which would also give the opportunity to significantly compare quantitative results with the qualitative data. As such, in the present study based on qualitative investigations, physiological and psychological measures are indicative only of magnitude.

#### Cortisol sampling and assays

Saliva was collected twice, by passive drool method, in small polypropylene tubes. Samples were immediately stored at -18°C, before further analyses. Enzyme-linked immunosorbent assay kits (Expanded Range High Sensitivity Salivary Cortisol Enzyme Immunoassay Kit No. 1–3002, Salimetrics, UK) were used to determine salivary cortisol levels. A 96-well microplate spectrophotometer (Multiska FC Microplate Photometer, Thermo Fisher, Germany) was used to measure absorbance at 450nm. Each well, containing samples or cortisol standards and controls, was duplicated. Intra-assay precision was determined from the mean of replicates with a single microplate (coefficient of variation 4.8 ±1.2%). Inter-assay precision was determined from the mean of average duplicates based on the cortisol standards wells for calibration, provided in the ELISA kit, used in separate runs (coefficient of variation 5.4 ±3.6%), but prepared from the same stock solution and dilutions. The concentrations of unknown samples, expressed in nmol/L, were computed by interpolation using a 4-parameter non-linear regression curve fit, as recommended by Salimetrics’ protocol.

#### Self-reported measures

Cognitive and somatic anxiety were assessed by the two corresponding subscales of the French version of the Competitive State Anxiety Inventory 2 Revised (CSAI-2R [47]). In reaction to perceived threat, cognitive anxiety refers to a cognitive form of apprehension that is negatively toned, whereas somatic anxiety is linked with physiological reactions consecutive to the activation of the autonomous nervous system. Participants rated the intensity of each item on a scale of 1 (not at all) to 4 (very much so) to measure cognitive anxiety (e.g., *“I am worried about losing”*) and somatic anxiety (e.g., *“My body feels tight”*). Internal consistency was satisfactory for cognitive anxiety in the post-stress (*α* = .84) and the pre-stress (*α* = .81), and for somatic anxiety in the post-stress (*α* = .74) and the pre-stress (*α* = .66), albeit somewhat weak for the former variable and rather questionable.

Emotional states (pleasure, arousal, and dominance) were assessed with the Self-Assessment Manikin (SAM [48]), a nonverbal pictorial assessment technique. Each dimension was scored on a scale with nine figures/levels. For the pleasure dimension, participants circled a figure ranging from a smiling figure to a frowning figure. A high score indicated a high level of pleasure. For the arousal dimension, participants circled a figure ranging from a large figure (excited) to a small figure (relaxed). A high score indicated a high level of arousal. Finally, for the dominance dimension, participants circled a figure from a large figure (maximum control of their emotions) to a small figure (lack of control). A high score indicated a high level of control. This scale had already been used with the TSST [49].

### Qualitative data collection

Two types of data were collected: audiovisual recordings and enactive interviews. Recordings were collected by an audio-video camera with a wide-angle lens positioned to the side of the room during all the TSST or the TSST-G. We used a wide shot to see the participants (the one in the TSST; all of them in the TSST-G), the two committee members, and all the tools of the protocols. These data were used to collect as many elements of participants’ activity as possible.

During the 18 enactive interviews, the participant was confronted with his own audio-video record [21,22]. Numerous recent qualitative studies have demonstrated the fruitfulness of this method for studying the dynamic of experience in the actor (e.g. [50–52]) and for studying emotion and stress [32–34]. The interview was very directive so as to provoke the re-emergence of elements of past experience when the participant was confronted with his own video recording. All the interviews were conducted by the first author of this paper, who had extensive experience of qualitative research and, specifically, of using enactive interviewing techniques. The interviews lasted approximately 40 min for the TSST and 70 min for the TSST-G to allow the re-emergence of the dynamic of all the past experience encountered during the 15-min TSST and the 25-min TSST-G. To understand the dynamic of the experience, the video was shown from the beginning of the TSST (or the TSST-G). The participant could see his own actions and verbalizations and the protocol environment with the two committee members. Whenever he wished, the participant could pause the video to describe, comment on, and show his own lived experience step by step, which in our study concerned his perceptions (e.g., sensations, sentiments, feelings, informational variables such as visual, kinesthetic, acoustic variables), intentions, and actions. The participant did not know the aim of the experiment. Before each interview, the rater explained the expectation that the participant should “re-live” and describe his own experience during the TSST (or the TSST-G), without *a posteriori* analysis, rationalization, or justification, as suggested for phenomenological research [53]. In order to eliminate pre-formed experiences, the participant was involved in an attitude of evocation. He was directed to avoid a theoretical description of his action but to evoke what he had experienced during the specific moment on video. Behavior indicators like hesitations in the stream of language, unstructured sentences or an introspective stare were synonyms of evocation [54]. Nevertheless, the experimenter’s questions did not directly evoke stress or emotion, to avoid influencing the expression of the participant’s feelings. The starting question was: “What are you doing here (pointing to the video image)?”, then: “What do you perceive at this moment?”, “What are your intentions?” According to the participant’s response, the questioning went deeper starting from the participant’s evocation. If a participant emphasized an emotional feeling during the interview, the principle of in-depth qualitative research dictated that the researchers investigate a more explicit and authentic report of experience, always in relation to the unfolding situation.

### Data analysis

Salivary cortisol levels, cognitive anxiety, somatic anxiety, pleasure, arousal, and dominance scores were analyzed for the TSST and the TSST-G with paired-sample *t* tests to evidence possible effects of time (pre-treatment, post-treatment) for each condition. Level of significance was defined as *p* < .05. Cohen’s *d* was used to calculate effect sizes. Statistical analyses were performed by Statistica 12 for PC.

High and low cortisol responders were selected according to the delta of their cortisol levels between the beginning and the end of the TSST and the TSST-G. The four participants selected for case studies were those who had the highest and the lowest delta between pre- and post-treatment: the two highest participants for the TSST and the TSST-G; the two lowest participants for the TSST and the TSST-G.

Qualitative data collection was analyzed following the phenomenological method, which articulates the inductive and deductive approaches by identifying the description of phenomena that can be clustered into discrete categories; taken together, these describe the center and the structure of the experience [53]. This phenomenological data analysis was conducted in seven steps following the custom of this theoretical and methodological framework [21,22].

First, the enactive interviews were transcribed verbatim and related to the chronological description of actions and communications using audio-video records.

Secondly, components of the experience were identified using the verbatims associated with the chronological description: the elementary units of meaning (which are the smallest units of activity that are meaningful for an actor?); perception (what sensations, sentiments, feelings are significant in the situation?), action (what do the participants do?), and intention (what concerns emerge within the action?).

Thirdly, these units of meaning were grouped into sequences that referred to the same story during the TSST and the TSST-G. These sequences are categorized within the temporality of the significant experience. This step allows us to describe and understand the dynamic of emerging experience.

Fourthly, typical experience was identified for each participant. Typicality corresponds to four aspects [55]: descriptive (i.e., the typical occurrence presents the highest number of traits of the experience analyzed in the sample of participants and the situations studied), statistical (i.e., the typical occurrence is the one most frequently observed in the sample studied), generative (i.e., the typical occurrence has a propensity to recur when conditions resembling those observed are reproduced), and significant (i.e., actors express a feeling of typicality when they are questioned about it during enactive interview). Thereby, each typical component of the experience was identified for each of the 18 participants: typical perception, typical action, and typical intention.

Fifthly, typical experience for all the participants was identified by comparing all the individual typical experiences. Data were compared to keep only the typical component of the experience recurrent for all.

Sixthly, we constructed the tables of typical live experience of the nine participants of both the TSST and the TSST-G. This construction crossed the preceding steps associating temporality, typical sequences, typical perceptions, typical actions, and typical intentions. For greater clarity, we normalized the formulation of the content of each category succinctly. The typical sequence was expressed shortly with one word or a group of words to describe qualitatively the specificity of the meaningful experience. Each component of the experience was reported using emblematic words of the participant. The typical perception expressed what all participants felt and perceived during a specific sequence. The typical action was normalized with the “ing” form expressing the ongoing action. The typical intentions were expressed starting with an infinitive verb to translate the dynamic state of the participants' common concerns.

Case studies were constructed in two steps for the four lowest and highest cortisol responders. The components of the experience were identified using verbatims associated with the chronological description (see second step of the preceding protocol). Then, the story of the experience was constructed for each participant during the TSST and the TSST-G. The story presented different components of the experience like actions, perceptions, and intentions in time order. Extracts of the enactive interview were used to give precise illustration.

Then, we compared tables of the typical experience of the nine participants of the TSST with the table of the TSST-G to identify the common and singular experiences between these two protocols.

## Results

### Preliminary results

A series of paired-sample *t* tests evidenced that cortisol levels, cognitive anxiety, somatic anxiety, and arousal were significantly higher at the end of the TSST or the TSST-G than in the beginning of these two stressful tasks, and that pleasure and dominance were significantly lower. Table 1 summarizes the descriptive statistics of cortisol levels, cognitive anxiety, somatic anxiety, pleasure, arousal, and dominance scores for both the TSST and the TSST-G conditions. Table 2 summarizes the results of the paired-sample *t* tests conducted on the previous variables from the beginning to the end of the TSST or the TSST-G.

**Table 1:** Descriptive statistics of physiological and psychological measures.

	TSST		TSST-G	
	Pre-stress (*M* and *SD*)	Post-stress (*M* and *SD*)	Pre-stress (*M* and *SD*)	Post-stress (*M* and *SD*)
**Salivary cortisol (nmol/L)**	7.83 ± 1.55	11.24 ± 2.97	7.73 ± 4.47	11.45 ± 7.02
**Cognitive anxiety**	1.36 ± 0.44	2.22 ± 1.07	1.80 ± 0.63	2.33 ± 0.73
**Somatic anxiety**	1.30 ± 0.32	1.78 ± 0.51	1.19 ± 0.18	1.76 ± 0.37
**Pleasure**	7.11 ± 1.27	5.33 ± 1.58	7.00 ± 1.73	5.33 ± 1.41
**Arousal**	2.89 ± 1.76	4.44 ± 2.07	3.11 ± 1.83	5.33 ± 1.73
**Dominance**	7.33 ± 1.00	5.33 ± 1.73	7.22 ± 1.30	5.67 ± 2.00

*M* = Mean, *SD* = Standard Deviation

**Table 2:** Results of the paired-sample *t* tests.

	TSST				TSST-G			
	*t*	*df*	*p*	Cohen’s d	*t*	*df*	*p*	Cohen’s d
**Salivary cortisol**	4.71	8	.002	1.44	3.08	8	.016	0.63
**Cognitive anxiety**	3.33	8	.010	1.05	6.05	8	< .001	0.78
**Somatic anxiety**	3.59	8	.007	1.13	4.54	8	.002	1.96
**Pleasure**	-2.40	8	.043	1.24	-9.43	8	< .001	1.06
**Arousal**	2.68	8	.028	0.81	15.12	8	< .001	1.25
**Dominance**	-3.00	8	.017	1.42	-3.28	8	.011	0.92

### Meaningful experience

In this section, we examine the experience descriptions corresponding to perceptions, actions, and intentions of the TSST and the TSST-G participants.

#### Typical experiences during the TSST and the TSST-G

We present in the following tables the typical experiences of the nine participants during the TSST (Table 3) and the TSST-G (Table 4). The experiences reported are considered typical because they are characteristic of the activity of all the participants. These experiences are described according to the time course of the TSST or the TSST-G (“Situation” column), categorized in sequences corresponding to the typical temporal experience of the actors (“Sequence” column), and presented according to the typical significations described in the form of perceptions, actions, and intentions.

**Table 3:** Typical experience of the nine participants of the TSST.

	Situation	SEQUENCES	Perceptions	Actions	Intentions
**Job interview task**	Entering the room Explanation of instructions	Exploratory investigation	Surprise Generally tense atmosphere	I’m listening I’m observing	Try to understand
	Preparation phase 3 minutes	Preparation	Time pressure Preparing	I’m organizing my presentation	Find and select ideas Keep within the time
	Interview phase 5 minutes	1) Recitation	Reassured to be doing what is planned	I’m reciting	Repeat the prepared introduction
		2) Expanding on ideas	Stressed by hesitations and memory lapses	I’m expanding on the ideas I’ve planned	Retrieve and state the ideas I have prepared Convince the committee
		3) Time pressure	Anxiety on seeing how much time is left	I’m watching the countdown	Know how far I’ve got
		4) Loss of control: Nothing more to say	Feel observed, judged Uncomfortable about saying whatever comes into my head Time perceived as interminable	I can’t think any more I’m searching chaotically I’m hesitating, I’m stuttering, I’m leaving gaps I’m constantly glancing at the time left	Fill the time Avoid gaps Look for support
		5) Abandon, resignation	Sense of incompetence Sense of looking incompetent	I’m losing my grip I’m doing nothing, I’m keeping quiet	Wait for it to end
		6) Detachment	Realize it’s not important	I’m laughing I’m laughing at myself	Try to reassure myself
		7) Paradoxical liberation	Hear beep at end of countdown Relief Sense of failure	I’m relaxing I’m staying concentrated for the second task	Self-evaluate Worry about what the committee will think of me
**Mental arithmetic task**	Explanation of instructions	Exploratory investigation	Pressure increasing Sense of relative easiness	I’m listening to the instructions	Try to understand
	Calculation phase 5 minutes	1) Calculation disturbed	Under time pressure Ashamed of making mistakes in an easy task	I’m going fast but I’m making lots of mistakes I’m getting lost: I’m recalculating several times	Not make mistakes Be quick
		2) Negative Spiral	Feel everything getting jumbled up in my head Aware of my negative thoughts	I can’t count any more I’m making repeated errors	Not look incompetent
		3) Seeking solutions	Withdraw into self	I’m looking for strategies	Find solutions Not lose face
		4) Abandonment, resignation	Fed up, I’ve had enough of this Realize it’s too much for me	I have nothing more to say I’m saying whatever comes into my head	Make the time go by
		2´) Repeated successes	Only thinking about the numbers	I’m concentrating I’m giving one right answer after another	Not make a mistake and have to go back to the start
		3) Paradoxical liberation	Hear beep at end of countdown Relief Sense of failure	I’m relaxing	Self-evaluate Leave

**Table 4:** Typical experience of the nine participants of the TSST-G.

	Situation	SEQUENCES	Perceptions	Actions	Intentions
**Job interview task**	Entering the room Instructions explained	Exploratory investigation	Surprise Generally tense atmosphere Embarrassed having to talk in presence of others	I’m listening I’m observing	Try to understand Worry about what the others will think of me
	Preparation phase 3 minutes	Preparation	Time pressure Reassured to be preparing	I’m organizing my presentation	Find and select convincing ideas
	Others performing	Alternation -Phases of exploratory listening -Phases of isolation	Stressed by long unpredictable wait for my turn Destabilized or reassured by others’ performances	I’m listening to parts of the speeches I’m putting myself in my bubble to repeat my speech	Find and select ideas Keep within the time
	Interview phase 2 minutes	Pressure spike	Hear my number Peak stress: I don’t remember anything	I’m looking at my number I’m repositioning	Not make a fool of myself
		1) Recitation	Reassured to be doing what was planned	I’m reciting	Repeat the prepared introduction
		2) Expanding on ideas	Stressed by hesitations and things forgotten	I’m expanding on the ideas I planned	Retrieve and state the ideas I have prepared Convince the committee
		3) Time pressure	Anxious at seeing the time left	I’m watching the countdown	Know how far I’ve got
		4) Loss of control: Nothing more to say	Uneasy at saying whatever comes into my head Feel observed, judged	I can’t think any more I’m searching chaotically I’m hesitating, I’m stuttering, I’m leaving silences I’m constantly glancing at the time left	Fill the time Avoid gaps Look for support
		5) Paradoxical liberation	Hear beep at end of countdown Relief Sense of failure	I’m relaxing I’m staying concentrated for the second task	Self-evaluate Worry about what the others will think of me
	Others performing	Alternation -Phases of comparative, empathetic listening -Phases of relaxation	Relaxation Feeling a “knot in the stomach”	I’m comparing what is being said with my own performance I’m sympathizing with my struggling peers I’m gloating I’m thinking of something else	Compare myself, try to reassure myself Relax a bit
**Mental arithmetic task**	Explanation of instructions	Exploratory investigation	Pressure rising Sense of relative easiness Stressed by having to calculate in front of everyone	I’m listening to the instructions	Try to understand
	Others performing	Training	Stressed by unpredictable wait for my turn Destabilized by others’ performances	I’m trying to do the sums I’m assessing the difficulty of the task	Get ready for my turn
	Calculation phase 80 seconds	Pressure spike	Hear my number Stress spike	I’m looking at my number I’m repositioning	Not make a fool of myself
		1) Calculation disturbed	Under time pressure Ashamed of making mistakes in an easy task	I’m going fast but I’m making a lot of mistakes I’m getting lost: I’m recalculating several times	Not make mistakes Be quick
		2) Negative spiral	Feel everything getting jumbled up in my head Aware of my negative thoughts	I can’t count any more I’m making repeated mistakes	Not look incompetent
		2´) Repeated successes	Only thinking about the numbers	I’m concentrating I’m giving one right answer after another	Not make a mistake and have to go back to the start
		3) Paradoxical liberation	Hear beep at end of countdown Relief Sense of failure	I’m relaxing	Self-evaluate Leave
	Others performing	Alternation -Phases of comparative, empathetic listening -Phases of relaxation	Relaxation Feeling a “knot in the stomach”	I’m comparing what is being said with my performance I’m feeling sorry for my struggling peers I’m gloating I’m thinking of something else	Compare myself, try to reassure myself Relax a bit
		End of task	Relieved but tense and disappointed	I’m relaxing	Leave

#### Common experiences between the TSST and the TSST-G

In this section, we focus on the typical experiences common to all 18 participants in the TSST and the TSST-G according to the time course of each protocol.

**Preparation period:** The “Exploratory investigation” is the first typical sequence of the participants’ activity when they enter the experiment room for the TSST and the TSST-G and when they listen to the instructions. In this sequence, the participant’s intention is to understand what is happening: Why is he here? What will he have to do? He investigates by observing his environment and listening carefully to the instructions. He is especially surprised and destabilized by the cold and unusual environment (the committee members in white lab coats, the camera, the long countdown on the timer) and the very formal character of the announcements (dry tone of voice, severe gaze of the two committee members). This investigative sequence is followed by a “Preparation” sequence in which the participant tries to reassure himself by organizing his job interview in the three minutes’ preparation time. His main intention is to find convincing ideas. This systematically gives rise to three types of action: (1) finding the job on which he will make his presentation; (2) listing ideas related to this job and his past experience; and (3) preparing for his interview. This involved constructing the introductory sentences, organizing his ideas by outlining a plan, and repeating it in his head. The time devoted to each type of action varies according to the participant. Some participants spend so much time finding the job that they have no time to prepare their presentation. This sequence is broadly reassuring for the participant because he is relieved to find things to say and so can retake control of the task.

**Mock job interview:** The participants’ progression through the job interview is divided into several chronological sequences. Five of them are common to the TSST and the TSST-G. The first sequence is “Recitation”, in which the participant recites what he has planned to say (generally a two-sentence introduction). He is in his bubble, he can speak without thinking, so his apprehensions are relieved. The second sequence experienced in the interview is “Development of ideas”. The participant’s intention is to retrieve and develop all the ideas worked out in the three minutes’ preparation. He wants to be convincing for the committee members. This sequence is experienced as stressful only when the participants are lost for words or forget ideas. The third sequence corresponds to the appearance of “Time pressure”. The participant’s intention is to see where has reached after having developed all his ideas. This is when he systematically watches the timer and realizes that he still has a lot of time left. This perception immediately gives rise to anxiety in the participant, who wonders how he will keep going for the remaining time. Even if it only lasts a fraction of a second, this sequence is experienced as very significant and structuring for the participant’s activity. The fourth sequence, “Loss of control” follows immediately. The participant’s intention is to fill the gap in order to reach the end of the allotted time. It is more important to keep going than to find convincing ideas. This period is marked by many hesitations, stuttering, and breaks. The participant explains that he cannot think any more. He is reduced to repeating ideas already presented, lying, inventing, talking about things that are not connected with the chosen job. These various actions make him uneasy, he feels observed and judged, so much so that he is very likely to stare at the wall so as not to have to look the committee members in the eyes. He constantly watches the time, which is experienced as interminable, as an enemy. This sequence is the most uncomfortable, fraught with a sense of failure and guilt. The final interview sequence common to the TSST and the TSST-G is “Paradoxical liberation”. This is the moment when the timer sounds at the end of this first task. The participant’s intention is to self-evaluate, because he is preoccupied with knowing what impression he made. His feelings are bivalent, divided between a sense of relief related to the end of the task and a sense of failure regarding his performance.

**Mental arithmetic task:** Following the job interview task, the mental arithmetic task also contains five typical sequences common to the 18 participants in the TSST and the TSST-G. The first sequence of the task is “Exploratory investigation”. This is very similar to the exploratory investigation carried out in the first task. The participant’s intention is always to understand what is happening. He is always attentive to the instructions, trying to anticipate what he will be asked to do. But he did not expect to be asked to do this kind of task and is surprised by the number 17 to be subtracted. This arithmetical challenge is first perceived as easy, then as increasingly difficult. The task is divided into several chronological sequences. Four are common to the TSST and the TSST-G. The first sequence is called “Calculation disturbed”. The participant’s intention is twofold: not to make mistakes, and to be quick. Under time pressure, he makes mistakes. He is ashamed of them, because he thinks the task should be easy. Two types of sequence may follow, depending on success or failure. In the case of failure, the participant falls into a sequence of “Negative spiral”. His intention is to avoid being seen as incompetent. He makes more and more mistakes and cannot calculate any more. He is in a negative spiral in which negative feelings hinder his thinking, for example: *“I cannot even do a simple subtraction*, *it’s driving me crazy”*, and/or *“Same mistake as just before*, *I’m an idiot*!*”*, (interview extracts). Such thoughts make him incapable. In the case of success, the participant enters into a sequence of “Repeated successes”. His intention is not to make a mistake and have to go back to the starting number. The participant is concentrating, inside his bubble, giving one right answer after another. His perceptions are entirely focused on the calculations. The final sequence is that of “Paradoxical liberation”. It corresponds to perception of the end of the task. As in the equivalent sequence in the job interview task, the participant’s feelings are bivalent, divided between relief and a sense of failure. This sense of failure is also felt by candidates who were on the whole successful, because they think they could have done better. The participant’s intention in this sequence of “Paradoxical liberation” is both to self-evaluate and to leave the experimentation.

#### Singular experiences between the TSST and the TSST-G

In this section, we focus on participants’ typical experiences which differ between the TSST and the TSST-G. Three important typical experiences can be distinguished at the levels of stress fluctuation, temporality, and the presence of peers.

**Stress fluctuation:** The participants do not experience the same stress fluctuation in the TSST and the TSST-G. The TSST-G participants experience a stress that varies much more throughout the protocol than that of the TSST participants. In the TSST-G, the waiting periods are experienced as stressful, especially the moments when a participant’s number is called out. The random order creates stress spikes before each call. Other participants’ performances also induce stress fluctuation. These periods are experienced as destabilizing when: (1) others give a performance that the participant considers of high quality, because he is not sure he can perform as well; (2) others are struggling, because the participant also now finds the task difficult, or feels empathy; (3) the participant is distracted by the sound of others’ performances and is not able to practice. Others’ performances may also be experienced as reassuring when: (1) others do not succeed, because the participant realizes he is not the only one to fail; (2) others are struggling, because the participant can gloat to relax; (3) his own performance is over, because he can relax and does not necessarily attend any more to what the others are saying.

**Temporality:** The analysis of experiences in the two protocols, the TSST and the TSST-G, reveals a difference at the level of temporality. Test durations are longer for the TSST, with 5 minutes’ job interview (vs 2 minutes for the TSST-G) and 5 minutes’ mental arithmetic (1 minute 20 seconds for the TSST-G). This time difference gives rise to a difference in the experiential processes, with two sequences that only appear in the TSST (see Tables 3 and 4). In the interview phase, the two sequences specific to the TSST are “Abandonment” and “Detachment”. The “Abandonment” sequence follows the “Loss of control” sequence. The participant is at the end of his tether, he cannot cope any more, he is resigned, and his intention is therefore to wait for the time to end without doing anything. He feels a strong sense of incompetence vis-à-vis the committee members and vis-à-vis himself. This sequence corresponds to intense negative emotions. Likewise, the “Detachment” sequence was only observed in the TSST; it comes after the “Abandonment” sequence. The participant’s intention is to reassure himself. He turns his performance into self-mockery. He tries to convince himself that the task is not all that important. Two additional sequences also appear in the mental arithmetic phase only for the TSST. The sequence called “Seeking solutions” follows the “Negative spiral” sequence. The participant’s intention is to find solutions so as not to lose face. He looks for strategies to succeed or protect himself; these strategies vary from one participant to another, for example, *“stop looking at the experimenters so as to avoid pressure”*, and/or *“work out the sums aloud”*. The “Abandonment” sequence in the arithmetic task is also specific to the TSST. The participant’s intention is make the time go by. He decides to say nothing any more or to give random answers. He perceives the task as insurmountable, he is irritated, he just wants it to end. The two additional sequences in each task are set in a temporal dynamic, they require more time and an accumulation of negative experiences. This may explain their absence in the TSST-G, in which the interview only lasts 2 minutes and the arithmetic test only 1 minute 20 seconds.

**The presence of peers:** The presence of peers, specific to the TSST-G, has a strong influence on the participant’s experience. The TSST-G protocol has six participants tested at the same time, whereas the TSST tests one participant at a time. At the experiential level, this difference between the TSST and the TSST-G reappears in four sequences–in the two “Exploratory investigations” and in the two “Paradoxical liberation” phases. In the “Exploratory investigations”, the actors are aware of the others in their environment even without seeing them; they reported being *“embarrassed at having to talk alone in the presence of the others”*, and/or *“having to do arithmetic in front of everyone”*. This affects the actors’ intentions even from the start of the experimentation, when they are preoccupied by what the others will think of them. Likewise, in the “Paradoxical liberation” sequences, the actors are preoccupied by what the others may have thought of them; their intention is to self-evaluate by comparing their performance with that of the others. This process is also present while the others are performing the requested task. By contrast, during their own interview and mental arithmetic phases, they report that they thought less about the other participants and mainly experience the threat of social evaluation by the committee members. So it can be seen that the presence of peers induces a comparison and social pressure specific to the TSST-G, since the TSST is conducted with only one participant each time.

#### Case studies of high cortisol responders

The results of the case studies of the low cortisol responders (lowest cortisol difference) did not indicate experiences significantly different from those of the other participants. These case studies are therefore not presented here.

The results of the case studies of the high cortisol responders (highest cortisol difference) are emblematic of the lived experience of these stressing experiments. We present in detail the stress dynamic of these two case studies for the highest cortisol responder during the TSST and the highest cortisol responder during the TSST-G.

#### TSST highest cortisol responder

**Fabrice:** The results show that Fabrice experiences increasingly intense stress during the TSST experiment. His initial intention to do the best he can progressively veers toward not losing face, then leaving. He finds the procedure increasingly oppressive, especially the big timer showing the time passing ever more slowly, and the stern-faced committee progressively judging his growing incompetence. He first thought the tasks easy but as he goes on he finds them harder and harder.

As soon as he enters the test room, Fabrice is surprised; he had not expected to find an empty room with two committee members in white coats sitting behind a table with a big timer. He tries to understand what they want him to do. He is relieved not to have to sit on the floor. Then the explanation of the job interview task puts him under pressure. The experimenters speak curtly; he thinks to himself: “This is serious, no joke at all, I can’t expect any help from them…. It’s not easy, because I’m on my own…”. Being able to take notes in the preparation time reassures him, because he can write down ideas. But when he realizes he will not be allowed to use his notes, he is destabilized: it is going to be harder than he thought. At this point, his intention is to give the best interview he can and hold out for the five minutes.

During the preparation phase, he hurries to choose a job, and the first one that comes to mind is that of postman. That will be original, no one will have thought of that. As the preparation goes on, the pressure mounts. He wonders if he can keep going for five minutes.

When he is called, his intention is to make a good impression. He adopts the appropriate posture for a job interview, all the more so because the committee has told him his posture will be analyzed. He starts his presentation by saying what he had planned. But very quickly he has finished and is fishing for ideas. As he speaks, he realizes that what he is saying is unstructured and his arguments are not very convincing. The pressure increases and he begins to lose control, as the following interview extract shows:

Fabrice: “I’m becoming more and more aware that it’s hard… er… As it goes on, I lose confidence. My aim now is to use up the time, whereas at the start it was to find good ideas. I’m becoming more and more stressed, although when I arrived I was relaxed!”

His intention is to fill the void. He is afraid of gaps, of being left with nothing to say in front of the committee, not being able to say anything. He is not pleased with himself, because he realizes he has nothing more to say when he is not even halfway through the time. From then on, he only looks at the timer to watch the countdown and avoid the committee’s inquisitorial gaze. His stress is increasing and the time is going by more and more slowly, as he experiences it. He is afraid of failure. He can sense that he is failing in the task he has been set. Negative ideas pile up in his head, he can feel the committee judging him. He cannot overcome this unease. He falls into a vicious circle, as this extract shows:

Fabrice: “I’m failing. I realize that my sentences are becoming meaningless. That’s what makes feel ill at ease. That, and the fact that I’m aware of it, stress me, and the stress amplifies the feeling and more and more I’m saying just anything.”

He just wants it to end. He is silent for a long time, thinks he is useless, and this prevents him from thinking. In his incapacity, he begins to move away from the instructions, and starts to laugh, saying he has “lost the plot.” Faced with this unease, he tries to reassure himself by telling himself it is not serious, it is not a real interview, it is not an evaluation, just an experiment. The final beep comes at last, relieving him but leaving a bitter taste.

In the mental arithmetic test, he is not confident at all. During the explanations, he wonders what will be asked for next. He thinks he will be questioned on numbers with several thousands. He is anxious. He walks away feeling defeatist, believing he will never manage. When he starts, he feels he is being judged. He is destabilized when the committee tells him: “That’s wrong, please start over from…”, “Faster, please.” Every interruption makes him lose the thread of his calculation. He is very hesitant. He gives a correct answer but thinks it deplorable because he has taken so long to find it. He falls into a vicious spiral in which negative images invade his thoughts, as the following extract shows.

Fabrice: “I’m coping less and less well. I’m thinking of loads of things and can’t concentrate on the sums… I can’t concentrate… I tell myself, ‘I’m useless, what will they think of me? … I can’t even subtract 17, and I’ve failed my interview, and arithmetic is not my thing, and I don’t want to be doing this….’ A real panic, in other words! … and then I just can’t think any more.”

Now he tries to relax, telling himself that if he carries on thinking that way he will never do it. He applies strategies, seeking to relax his body, avoiding the gaze of the committee, who are a stress factor for him, trying to reassure himself that it cannot get worse than it is now. But he still cannot succeed, he feels shame and judges himself “useless”. It is even worse than before, two minutes have gone by and he has only given two answers: “It’s a disgrace to be so useless, I am not at all pleased with my performance.” His stress level is very high. When he hears the final beep, he gives a great sigh indicating an enormous relief. He is ashamed of seeming useless but delighted that it is over.

#### TSST-G highest cortisol responder

**François:** The results show that François is very affected by the TSST-G protocol; his main intention is to avoid making himself ridiculous in front of his peers and the committee. He experiences the experiment as very stressing, especially the environment (the partitions, the committee members in white coats, the timer), the atmosphere (the instruction not to talk with the others, who cannot even be seen, the curt tone and stress-inducing interruptions of the committee), the unknown order of testing, and his own performance (a sense of failure in both tasks). These feelings vary through the different stages of the experiment.

As soon as he enters the test room, François is destabilized by the environment of the experiment. He is struck by the presence of the partitions, the numbers on the floor, the two committee members, and the camera. His intention is to understand what is going to happen, as this interview extract shows:

François: “I say to myself ‘What have I got myself into here?’ I didn’t think it would be like that. I’m out of my… comfort zone. ‘What’s going to happen? … I’m in an observation phase… it’s weird…. A really strange situation for me. I don’t like being destabilized. I like to have things announced well in advance.”

When the job interview task instructions are given, François listens carefully so as to do what is asked of him as well as he can. He is struck by the very directive, curt tone of the committee members. He is not confident in this context, which he describes as “strange”.

During the preparation phase, François quickly writes down numerous notes about his qualities. He is very concerned to make a good impression, but increasingly uncomfortable because he does not know what job to choose.

Then he anxiously waits to see who will be called first. When he hears “Participant number 3!” he is relieved that he is not going first, since he will be able to see how it unfolds. He stands straight, with his hands behind his back, in his place, trying not to move, so as to give the committee a good impression. His relief comes to an end when he hears No. 3’s performance, which he judges to be good. He feels the pressure rising, especially at the end of the performance, because he wonders if it may be his turn next.

When his number is called, he feels a spike of pressure. He experiences negative emotions, as the interview extract shows:

François: “Then there’s a surge of pressure when I hear the number. And all at once I say to myself that it is going to be a disaster, because my pressure has suddenly gone up and since, er… I like things to be well-prepared.

Researcher: “Now, what was that pressure? What makes you feel under pressure at this point?”

François: “Well, I say to myself, ‘You’re not ready, at least not for this interview. . . .’ and also not ready to be compared with the others, I think, to be overheard by the others.”

Researcher: “Right, so you feel under pressure because of the other participants at this point?”

François: “Yes, and also because of the experimenters. Because I … it’s hard to explain… I don’t like not knowing how I will be seen by people I don’t know. And it comes down to not being prepared… well, for me it is a big failure all the same. I’m saying to myself, ‘What are they going to think of me?’ That unsettles me!”

François’s experience is marked by the presence of others; it adds to the pressure on him. He starts his presentation on how he would be a good football trainer, as he had planned on his piece of paper. But very soon he has used up all his ideas and starts to focus on the timer. He panics because there is a lot of time left with nothing more to say. He is completely destabilized; his main intention is now to fill up the time. He feels the gazes of the two experimenters as increasingly oppressive, so much so that he no longer even dares to look at them. The experimenters say: “There is still time left, carry on.” François smiles. He explains that this is a defensive strategy he uses in the interview.

François: “I’m trying to mask a little bit what I’m feeling, to indicate in my appearance that I’m still at ease… to show I’m not really stressed… it’s a job interview… but my brain is in turmoil. I’ve said it all and there’s nothing left to say. Try as I may, with the pressure and the stress, I can’t find words any more.”

François hears the beep marking the end of the interview: it is the end of the ordeal for him, he relaxes, with a strong sense of failure. While the others are interviewed, he looks at the floor, comparing himself with them. He is impressed by the ideas they come up with, and this makes him feel even more of a failure.

In the mental arithmetic task, he is again uncomfortable. He expects it to be another catastrophe for him, and this stresses him. He thinks he is weak in math and especially in mental arithmetic. When he listens to the other participants, he tries to calculate at the same time and watches the experimenters’ reactions. He finds them very cold, like robots. As the participants are called, in random order, he thinks each time it will be his turn, and this provokes small peaks of stress, each followed by relief that he is not the one.

When he hears his number, he is completely destabilized. He is stressed and cannot think at all. He wants to go away and hide. The experimenters’ interruptions (“That is wrong”, “Faster, please”) are experienced as particularly stressing. He cannot even work out the first numbers, and this irritates him. When his test comes to an end, he feels relieved even if he has poor self-esteem. While the remainder are tested, he continues to think over his failure, telling himself he has been really feeble. Then he starts listening to see how far they can get. When the TSST-G is over, he is relieved, but feels sick inside because of his poor performance and the image he has given of himself.

## Discussion

The aim of this study was to conduct qualitative investigations to examine in more depth the psychosocial stress experienced by participants confronted with the TSST or the TSST-G. The preliminary results first showed that cortisol levels, cognitive anxiety, somatic anxiety, and arousal significantly increased from the beginning to the end of the TSST and the TSST-G, whereas pleasure and dominance decreased. These results were in line with those of the literature (e.g. [11,15,17,18]), evidencing that social-evaluative threat and uncontrollability are indeed two stressful components when they are combined, according to the theoretical framework of psychological stress induced by the TSST and the TSST-G [10]. Despite the small sample size, the findings of the study showed reassuring consistencies with previous quantitative studies examining some of the physiological and psychological markers of the TSST and the TSST-G. Thanks to qualitative analyses, we can examine the meaningful experience of participants and compare it with social-evaluative threat and uncontrollability, which are theoretically the two stressful components of the TSST and the TSST-G. The case studies results are in line with typical experiences of the 18 participants during the TSST (Table 3) and the TSST-G (Table 4). The lowest cortisol responder case studies did not show anything significantly different from the other participants. The highest cortisol responder case studies experienced an important social-evaluative threat and uncontrollability. This threat is even more pronounced than the typical experience of the other participants, inducing higher cortisol levels. We can hypothesize that low and high cortisol responders had similar experiences to the others, but respectively less and more intense, or with a slower or faster dynamic. A wider study with more participants could investigate this hypothesis (see “Limitations and direction for future studies” section below), especially for low responders.

### Social-evaluative threat, different facets of a common theme

Social-evaluative threat occurs when an individual is or could be negatively judged by others. As expected, the social-evaluative threat induced by the two committee members in white lab coats, by their attitude throughout the experimentation, and by the video camera, was considered especially stress-inducing by the participants. This is a common theme of the experiences reported by the participants of the TSST and the TSST-G in qualitative analyses, regardless of which step of these procedures (preparation period, mock job interview, or mental arithmetic task). They even reported that they had developed strategies to avoid the experimenters’ evaluation. The two highest cortisol responders avoided looking at the committee members, because they felt judged. Fabrice focused his attention on the countdown, and François looked up. In the literature, the effects of the TSST and the TSST-G on different physiological and psychological markers were similar despite procedural differences (see also [11]). But while social-evaluative threat was previously described as particularly stressful by all the participants in qualitative analyses, this stress was also experienced differently between the TSST and the TSST-G.

In the TSST, social-evaluative threat was not experienced as a constant threat by the participants. This threat was immediate from the start of the 5-min preparation period, but had not yet reached its highest level. While social-evaluative threat was also present at the beginning of the mock job interview and the mental arithmetic task, participants declared that it increased very significantly when they began to find it difficult to provide relevant ideas in the mock job interview or right answers to the subtraction task, until a stress peak was reached, leading to abandonment. The duration of the two tasks constituting the TSST (2 x 5 min) compared with the TSST-G (2 min and 80 sec) led the participants to experience specific states, notably abandonment, in which social-evaluative threat reached its climax. While we expected that social-evaluative threat would be temporally relatively homogeneous throughout the TSST due to the continued presence of the two experimenters, the results of our study showed that self-evaluative threat increased in the course of the mock job interview and the mental arithmetic task, especially when participants encountered difficulties in meeting the demands of the task. This result is in line with the framework of the self-preservation theory [5], in which failure in front of others is considered a lack of competence or intelligence that is debilitating for social esteem and/or social status, leading to physiological (e.g., salivary cortisol) and psychological (e.g., anxiety, emotions) responses similar to those produced in the present study. This is why the participants developed protection strategies such as self-mockery or detachment to avoid showing their incompetence to themselves and the experimenters.

The perceived experience of social-evaluative threat in the TSST-G was quite different from that perceived in the TSST. The presence of peers made social-evaluative threat specific, although they could not see them because they were separated by mobile dividing walls. As soon as the instructions were given to the participants during the 3-min preparation period or before the beginning of the mental arithmetic task, they thought of the presence of the others. They were hampered by the presence of peers and were worried about what they would think of them. This result confirmed the specific social-evaluative threat induced by the TSST-G with both the committee members and the peers [11]. But the results of our study highlight two complementary advances toward a better understanding of the participants’ experience during the TSST-G. First, the stress experienced by the participant throughout the TSST-G was temporally dynamic because it depended on the performance of others, whether good or bad. Peers were therefore considered potential evaluative others, but also a stressful means of comparison. According to the qualitative analyses, this comparison may also be a way of trying to reassure oneself. In any case, participants want to maintain their social status relative to others and to produce, through comparison, psychobiological responses to psychosocial threats related to their social status [56]. While the participant was not performing the mock job interview or the mental arithmetic task, listening to another participant was nonetheless a direct threat to the goal of maintaining his social self through comparison of their respective performances, leading to negative self-evaluations and increases in cortisol [4]. Secondly, qualitative analyses surprisingly showed that participants thought about peers very much less when they were performing the mock job interview or the mental arithmetic task than when they were listening to the performances of other participants. When they performed these two tasks, social-evaluative threat was mainly represented by the two experimenters, who had their attention focused on the participant they had called. Because the committee members did not smile or nod, but stared at the participant, were presented as experts in nonverbal behavior analysis, and withheld any type of social engagement or positive feedback [10], social-evaluative threat was essentially induced by the two experimenters during the 2-min mock job interview and the 80-sec mental arithmetic task, while it was induced by both other participants and committee members during the other parts of the TSST-G. In sum, qualitative analyses showed that social-evaluative threat fluctuated throughout the stress procedure, evidencing a dynamic of the stress experience and that social-evaluative threat did not come from the same persons during the TSST-G, depending on the sequences of this stressful task.

### From temporary and illusory controllability to suffered uncontrollability

With social-evaluative threat, uncontrollability is one of the two main stressful components of the TSST and the TSST-G. In the theoretical framework, uncontrollability is included throughout the procedure [10]: the room is unknown to the participants, they are not aware of the tasks they will have to do, the duration of the preparation period is short, the mental arithmetic task is totally unanticipated, and the committee members are non-responsive to potential social interactions. Our results showed that the previous forms of uncontrollability did not induce the same level of stress among participants. Qualitative analyses evidenced that the unknown room, the attitude of the experimenters, and the fact that participants were not aware of the tasks were considered particularly stressful. Contrary to the previous hypothesis, the preparation period is mainly considered reassuring because participants were relieved to have things to say in the following mock job interview, and consequently this gave them a sense of being in control. This may explain why a shortened speech preparation time did not influence cortisol responses [40]. Concerning the unanticipated mental arithmetic task, the participants self-reported that they were indeed surprised when it was announced, leading to a form of uncontrollability. However, participants declared that they quickly regained control because they initially considered the subtraction task easy.

The literature evidences that the TSST and the TSST-G significantly led to a progressive lack of control. Qualitative analyses provided two main complementary results. First, what was really stressful for the participants was losing control of the tasks that they were expected to master. They initially thought that they were in control of the mock job interview thanks to the preparation period and the ideas they had developed during it. They also initially thought that they controlled the mental arithmetic task, because they considered a series of subtractions a simple task. But when they began to encounter difficulties in meeting the demands of the task and when they took a look at the big stopwatch with time still left, a stress peak was declared by the participants. Although it would be quite short, this sequence was considered by the participants to be one of the most meaningful and stressful sequences of the TSST and the TSST-G. It was at that point that the participants began to lose control of the situation until the end of the task, going so far as to abandon or to resign. In case study with Fabrice, the loss of control dynamic was very clear. At the beginning, he thought the tasks were easy, then progressively he was surprised at failing; he could not understand. At the end, he was saying “whatever came into his head”. He was all the more ashamed of himself, because he had initially considered the task easy. Dickerson and Kemeny [4] showed that uncontrollability induced cortisol responses only if an important goal was threatened, such as the social self. When a participant initially thought that he could control the proposed tasks (which tended to protect his social self), the experience was considered particularly stressful in this study because the uncontrollable conditions of the TSST and the TSST-G created an unexpected context of forced failure. We may conclude that uncontrollability is indeed a stressful component of the TSST and the TSST-G, but qualitative analyses showed that what was really stressful for the participants was precisely suffering uncontrollability after a sequence of temporary and illusory controllability. Moreover, qualitative analyses highlighted that during the TSST participants experienced sequences of abandonment, detachment, or resignation, which are consequences of uncontrollability. It is a form of learned helplessness, i.e. the failure to escape shock induced by uncontrollable aversive events, because passivity and heightened anxiety are the default reaction to prolonged bad events [57]. In sum, the uncontrollability experienced by the participants involved several stages throughout the TSST and the TSST-G: illusory controllability (“Exploratory investigation” sequence), discovery of uncontrollability (“Time pressure” and “Calculation disturbed” sequences), confirmation of uncontrollability (“Loss of control” and “Negative spiral” sequences), and even abandonment, specifically in the TSST (“Abandonment” and “Detachment” sequences).

Secondly, the procedure of the TSST-G leads the committee to call on each of the participants in random order to start their speech or to perform the mental arithmetic task [11]. This part of the procedure was not explicitly related with uncontrollability in the seminal work of von Dawans et al. [11]. However, participants declared in qualitative analyses that each moment before the calling of a participant’s number was a moment of acute stress. As seen previously, stress fluctuated throughout the TSST-G and the randomized calling strongly contributed to this fluctuation. The participants were faced with a typical form of uncontrollability in which they were unable to avoid negative consequences and their behavioral responses did not affect the outcome [4]. Nothing can be done to influence the announcement of the participant’s number, which further leads them to consider these announcements uncontrollable and consequently particularly stressful. It could be interesting to include in the TSST-G procedure a brief pause just before the announcement of the participant’s number so as to reinforce the uncontrollability dimension of the TSST-G and consequently the stress response.

According to the meta-analysis conducted by Dickerson and Kemeny [4], social-evaluative threat and uncontrollability were simultaneously necessary to induce the largest and the most reliable increases in cortisol in motivated performance tasks such as the TSST or the TSST-G. The pattern was similar with qualitative analyses, because participants declared that social-evaluative threat was at its highest level when they began to lose control and to make errors. Finally, the last sequence of the experience declared by participants was a “Paradoxical liberation”, in which they simultaneously felt relief that the tasks were completed and a sense of failure. The social-evaluative threat and the uncontrollability components of the psychosocial stress continued, whereas the stressor was stopped This could have contributed to the high levels of subjective stress and salivary cortisol in the point of measure immediately after the TSST or the TSST-G, even if significant correlations between cortisol responses and perceived emotional stress variables were found in a minority of studies [46].

### Limitations and directions for future studies

The present study is not without limitations. We have seen previously in the Methods section that the small sample size, the day-time sessions of the TSST and the TSST-G, and the choice of two measurement points to assess cortisol levels did not allow us to draw any statistical conclusions beyond the main effect of time identified in the preliminary results. The main purpose of the study was to conduct qualitative investigations of the participants’ experience during the TSST and the TSST-G. Consequently, the quantitative part of the study was carried out only to replicate previous findings and to show an effect of time across all measures, in order to conduct qualitative investigations on a stronger basis than only making qualitative investigations. While we examined the participants’ experience during the TSST or the TSST-G, future studies may explore participants’ experience during all the different steps of the stress response (anticipation, acute response, and recovery) with a bigger sample size and more numerous measurement points of cortisol [40]. This kind of study would give the opportunity to significantly compare quantitative results with the qualitative data. In addition, we can consider the possibility of including the Estimation of Affective States scale [33] to retrospectively estimate the positive or negative character of their experiences during the TSST or the TSST-G during the enactive interview to help conduct the interview and to give quantitative indicators. Immediately following this step, sentiments could be documented from enactive interviews [33]. Finally, other measures of psychosocial stress could be included in these future studies, including markers of the sympathetic nervous system activity such as salivary alpha amylase (e.g. [58]) or measures related with the cardiovascular system (e.g. [17]).

Secondly, it would have been interesting in this perspective to use a crossover design rather than a between-subjects design, often more powerful than a two-group comparison study. This would have enabled us to directly compare the experiences of the same participant during the counterbalanced TSST and TSST-G. Although interesting, this alternative was not chosen, because repeated exposure to stressful situations such as the TSST leads to a habituation of HPA stress axis responses [45]. Consequently, it would be difficult to compare the two conditions for the same participant, because the uncontrollability dimension of the TSST and the cortisol responses would have certainly decreased in the second stage.

Thirdly, we have presented some qualitative investigations with case studies by separating low and high responders according to their cortisol levels. Indeed, cortisol responses due to psychosocial stress may vary greatly depending on the individuals [3], which may influence different outcomes. For example, whereas no general effect of psychosocial stress was found on declarative memory processes, high cortisol responders displayed better immediate free recall after being exposed to stress [59]. In order to compare quantitative and qualitative data in future studies, increasing the sample size would also give the opportunity to capture high, low or non-responses of a larger number of participants and to investigate their experiences with qualitative methods beyond the case studies presented here. In this way, we may overcome the limited result of our study regarding low cortisol responders, or the specificity of the experience of high and low cortisol responders, and consequently understand a possible cause of the evolution of cortisol response.

Fourthly, the qualitative part of the present study is considered a retrospective study in which a phenomenon is studied by looking back at events that have already happened. Retrospective studies are not without limitations. For example, participants might forget, suppress, or fail to remember certain factors–it is difficult to separate real from perceived or putative causes; or a cause-and-effect relationship cannot be determined [60]. But retrospective studies are useful to provide an original point of view on theoretical and empirical questions, especially when validated methods are applied, such as the enactive phenomenological framework used in the present study.

Finally, non-stress control conditions (placebo) were created for the TSST [61] and the TSST-G [11]. The same principle is applied for both tasks. The aim of the placebo conditions is to control for orthostasis, effects of speech itself, and general cognitive load. Consequently, all dimensions of the TSST or the TSST-G are kept, but uncontrollability and social-evaluative threat are excluded. The results evidenced that there was no cortisol response and no significant self-reported anxiety in the placebo conditions. Furthermore, a friendly TSST (f-TSST) has recently been designed [62], which involved speaking in front of a committee about hobbies or a favorite film. The experimenters smile, encourage the participant, and relaunch the conversation. The f-TSST induced neither cortisol rise nor negative affect. Use of a qualitative approach conducted in the present study would be appropriate for a better understanding of the states experienced by the participants during these non-stressful tasks.

## Conclusion

The present study is the first to conduct qualitative investigations to further clarify the stressful experience of the participants during the TSST and the TSST-G. The TSST and the TSST-G effectively induced psychosocial stress, with cortisol levels, cognitive anxiety, somatic anxiety, and arousal increases, and with pleasure and dominance decreases. In accordance with the theoretical framework, qualitative analyses showed that psychosocial stress was actually induced by the combination of social-evaluative threat and uncontrollability experienced by the participants. But the results also showed (1) that psychosocial stress is a dynamic phenomenon, with important fluctuations throughout the procedures, especially for the TSST-G; and (2) that despite the similar physiological and psychological responses between the TSST and the TSST-G evidenced in the literature, the experience of the participants was both similar and specific. While quantitative analyses provide interesting pre-stress/post-stress comparisons certifying that the TSST and the TSST-G were indeed stress-inducing, qualitative analyses provide a complementary point of view to access the meaningful experience of participants during these stressful procedures and the dynamic of the subjective stress experience [35] without disrupting it. Improving our knowledge about individuals’ experience confronted with social-evaluative threat and uncontrollability has implications for understanding the impact of stress and psychological risks in multiple domains, such as education, sport, work, and neuropsychiatric diseases [10].
